# Retraction Note: Comprehensive investigations of mixed convection of Fe–ethylene-glycol nanofluid inside an enclosure with different obstacles using lattice Boltzmann method

**DOI:** 10.1038/s41598-025-11088-6

**Published:** 2025-07-15

**Authors:** Chenqi Fu, Amin Rahmani, Wanich Suksatan, S. M. Alizadeh, Majid Zarringhalam, Supat Chupradit, Davood Toghraie

**Affiliations:** 1 College of Engineering and Technology, Jilin Agricultural University, Changchun, 130118 China; 2 Department Mechanical Engineering, Isfahan University of Technology, 84156 Isfahan, Iran; 3 Faculty of Nursing, HRH Princess Chulabhorn College of Medical Science, Chulabhorn Royal Academy, Bangkok, 10210 Thailand; 4 Petroleum Engineering Department, Australian College of Kuwait, West Mishref, Kuwait; 5 Young Researchers and Elite Club, South Tehran Branch, Islamic Azad University, Tehran, Iran; 6 Department of Occupational Therapy, Faculty of Associated Medical Sciences, Chiang Mai University, Chiang Mai, 50200 Thailand; 7 Department of Mechanical Engineering, Khomeinishahr Branch, Islamic Azad University, Khomeinishahr, Iran

Retraction of: *Scientific Reports* 10.1038/s41598-021-00038-7, published online 20 October 2021

The Editors have retracted this Article.

After publication concerns were raised over the repeating coefficients in tables pertaining to Equations 13 and 14, and overlapping panels within Figure 3 in columns Ri - 0.1 and Ri = 1. Additionally, References 20-25, 38 and 39 were found either to be irrelevant or to not correspond to what is reported in the Article. The Authors did not provide a satisfactory explanation to these issues. The Editors therefore no longer have confidence in the contents of this Article.

Davood Toghraie did not explicitly state if he agrees or disagrees with this retraction. Chenqi Fu, Amin Rahmani, Wanich Suksatan, S. M. Alizadeh, Majid Zarringhalam, and Supat Chupradit did not respond to the correspondence from Editors about the retraction.

